# Supplementary material for: Contextual subspace variational quantum eigensolver calculation of the dissociation curve of molecular nitrogen on a superconducting quantum computer
Source: npj Quantum Inf. 2025 Feb 12;11(1):25. doi: 10.1038/s41534-024-00952-4 (PMC11821530; doi:10.1038/s41534-024-00952-4)
Supplement: Supplementary file 1 — Supplementary Information [file 41534_2024_952_MOESM1_ESM.pdf]

Supplementary Information: Contextual Subspace Variational Quantum Eigensolver Calculation of the Dissociation Curve of Molecular Nitrogen on a Superconducting Quantum Computer

Tim Weaving*,[1] Alexis Ralli,[1,2] Peter J. Love,[2,3] Sauro Succi,[4,5,6] and Peter V. Coveney[1,7,8]

[1] *Centre for Computational Science, Department of Chemistry, University College London, WC1H 0AJ, United Kingdom**

[2] *Department of Physics and Astronomy, Tufts University, Medford, MA 02155, USA*

[3] *Computational Science Initiative, Brookhaven National Laboratory, Upton, NY 11973, USA*

[4] *Center for Life Nano-Neuro Science @ La Sapienza, Italian Institute of Technology, 00161 Roma, Italy*

[5] *Department of Mechanical Engineering, University College London, WC1E 7JE, United Kingdom*

[6] *Department of Physics, Harvard University, Cambridge, MA 02138, USA*

[7] *Advanced Research Computing Centre, University College London, WC1H 0AJ, United Kingdom*

[8] *Informatics Institute, University of Amsterdam, Amsterdam, 1098 XH, Netherlands*

(Dated: August 20, 2024)

Here we provide Supplementary Information (SI) for the main text **Contextual Subspace Variational Quantum Eigensolver Calculation of the Dissociation Curve of Molecular Nitrogen on a Superconducting Quantum Computer**. This includes the following:

1. A representative summary of variational quantum eigensolver (VQE) experiments run on quantum hardware in the period $2013-2023$ (Section I);
2. The 5-qubit quantum circuits generated via hardware-aware ADAPT-VQE that were subsequently executed on IBM hardware to produce the results of the main text (Section II);
3. Various correlation diagnostics tools that are commonly used to quantify the electron correlation present in a molecular wavefunction, which supplements our new tool presented in the main text (Section III);
4. A comparison of STO-3G and STO-6G basis sets for the molecular nitrogen problem (Section IV);
5. A comparison of different active space selection methods, either around the Fermi level or using MP2/CCSD natural orbitals (Section V);
6. The explicit Pauli Hamiltonians whose energies where evaluated through VQE (Section VI).

* timothy.weaving.20@ucl.ac.uk

I. VQE EXPERIMENT HISTORY

Year	Reference	System(s)	Ansatz	Max qubits	Platform	Hardware Vendor
2013	Peruzzo *et al.* [1]	HeH^+	UCC	2	SP	In-house
2015	Shen *et al.* [2]	HeH^+	UCC	1 qudit	TI	In-house
2015	Google Quantum [3]	H_2	UCC	2	SC	Google
2016	Santagati *et al.* [4]	Chlorophyll pair	Parametrized Hamiltonian	2	SP	In-house
2017	Kandala *et al.* [5]	H_2, LiH, BeH_2	Hardware Efficient	6	SC	IBM
2017	Colless *et al.*[6]	H_2 (excited states)	Hardware Efficient	2	SC	In-house
2018	Hempel *et al.* [7]	H_2, LiH	UCC	3	TI	In-house
2018	Kandala *et al.* [8]	H_2, LiH (magnetism)	Hardware Efficient	4	SC	IBM
2019	Nam *et al.* [9]	H_2O	UCC	4	TI	IonQ
2019	Smart & Mazziotti [10]	H_3	custom	3	SC	IBM
2019	McCaskey *et al.* [11]	NaH, RbH, KH	UCC and Hardware Efficient	4	SC	IBM, Rigetti
2020	Rice *et al.* [12]	LiH (dipole moment)	Hardware Efficient	4	SC	IBM
2020	Google AI Quantum [13]	H_6, H_8, H_{10}, H_{12}, HNNH	Hartree-Fock	12	SC	Google
2020	Gao *et al.* [14]	PSPCz	R_y	2	SC	IBM
2021	Kawashima *et al.* [15]	H_{10}	qubit-CC	2	TI	IonQ
2021	Eddins*et al.* [16]	H_2O	Entanglement Forging	5	SC	IBM
2021	Yamamoto *et al.* [17]	Crystalline Iron Model	UCCSD-PBC	2	SC	IBM
2021	Kirsopp *et al.* [18]	Oxazine derivatives	YXXX	4	SC, TI	IBM, Quantinuum
2022	Huang *et al.* [19]	H_2, CO	Linear Response	4	SC	In-house
2022	Lolur *et al.* [20]	HeH^+, LiH	Hardware Efficient	4	SC	IBM
2022	Leyton-Ortega *et al.* [21]	H_2	UCCSD	4	SC	IBM
2022	Liang *et al.* [22]	H_2, HeH^+, LiH, H_2O, NaH, CO_2	NAPA	6	SC	IBM
2022	Motta *et al.* [23]	H_3S^+	Entanglement Forging	6	SC	IBM
2022	O'Brien *et al.* [24]	Cyclobutene Ring	upCCD	10	SC	Google
2022	Khan *et al.* [25]	CH_4	UCCSD	6	TI	Quantinuum
2022	Zhao *et al.* [26]	Li_2O	oo-upCCD	12	TI	IonQ
2022	Guo *et al.* [27]	H_2, LiH, F_2	UCCSD	12	SC	Zuchongzhi 2.0
2023	Weaving *et al.* [28]	HCl	Hardware Efficient	3	SC	IBM
2023	Liu *et al.* [29]	H_2, HeH^+	Hardware Efficient	1 qudit	SC	In-house
2023	Dimitrov *et al.* [30]	CH_3F	pUCCD	11	TI	IonQ
2023	Jones *et al.* [31]	H_2O	UCCD	8	SC	IBM
2023	Liang *et al.* [32]	NH, BeH^+, F_2	SpacePulse	6	SC	IBM
2023	Weaving *et al.* [This work]	N_2	Hardware-Aware ADAPT	5	SC	IBM

TABLE I: A decade of experimental realizations of VQE for quantum chemistry; the list is not exhaustive. The works are listed chronologically by the date of initial preprint availability, not the final publication date. The platform keys are silicon photonic (SP), superconducting (SC) and trapped-ion (TI).

II. ANSATZ CIRCUITS

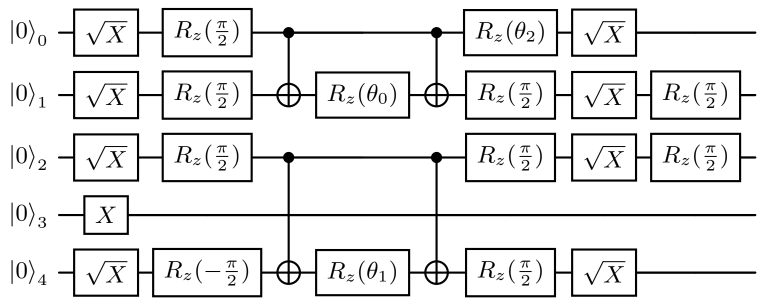

(a) $x = 0.8$Å; prepares $|\psi(\boldsymbol{\theta})\rangle = e^{i\theta_2 Y_0} e^{i\theta_1 Y_4 X_3} e^{i\theta_0 X_1 Y_0} |1_4 1_3 0_2 0_1 0_0\rangle$

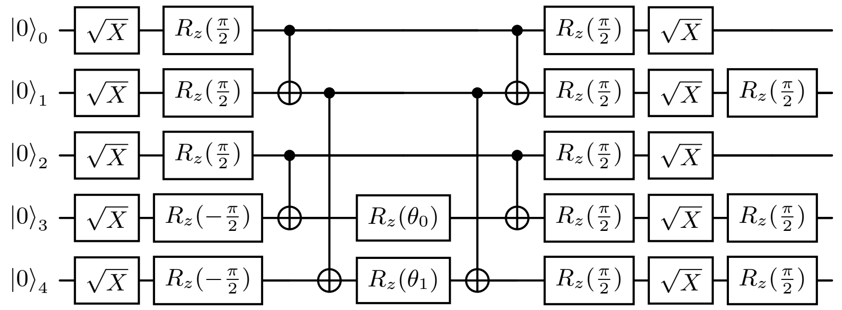

(b) $0.8 < x \leq 1.2$Å; prepares $|\psi(\boldsymbol{\theta})\rangle = e^{i\theta_1 Y_4 Y_1 Y_0} e^{i\theta_0 X_3 Y_2} |1_4 1_3 0_2 0_1 0_0\rangle$

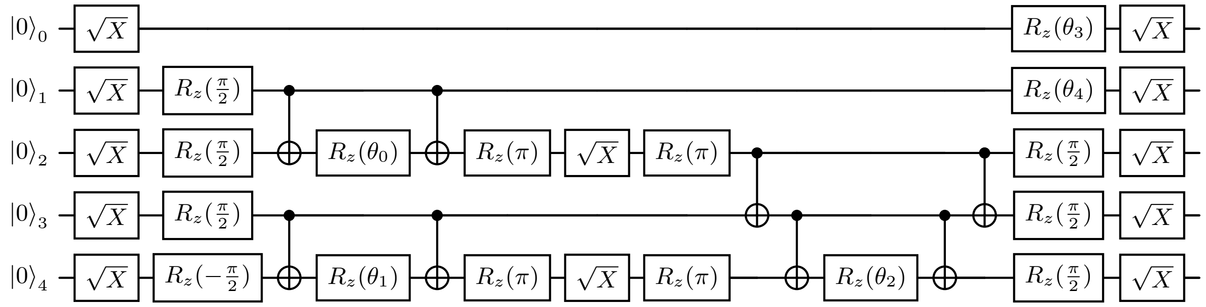

(c) $1.2 < x < 2.0$Å; prepares $|\psi(\boldsymbol{\theta})\rangle = e^{i\theta_4 Y_1} e^{i\theta_3 Y_0} e^{i\theta_2 Y_4 Y_3 Y_2} e^{i\theta_1 X_4 Y_3} e^{i\theta_0 X_2 Y_1} |1_4 0_3 0_2 0_1 0_0\rangle$

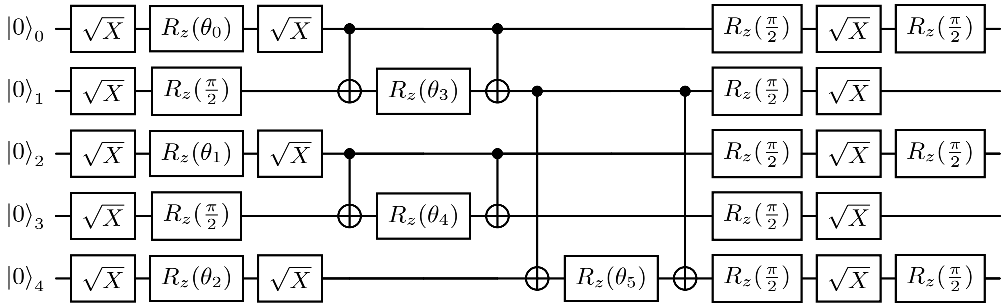

(d) $x = 2.0$Å; prepares $|\psi(\boldsymbol{\theta})\rangle = e^{i\theta_5 Y_1 X_4} e^{i\theta_4 Y_3 X_2} e^{i\theta_3 Y_1 X_0} e^{i\theta_2 Y_4} e^{i\theta_1 Y_2} e^{i\theta_0 Y_0} |0_4 0_3 0_2 0_1 0_0\rangle$

FIG. 1: Ansatz constructions for differing bond lengths $0.8 \leq x \leq 2.0$Å for the N_2 simulation in the Results section of the main text, expressed in the IBM Quantum native gate-set. Excitations were selected using Hardware-Aware ADAPT-VQE, described in the Methods section of the main text, and the resulting circuits were heavily optimized for compactness.

III. CORRELATION DIAGNOSTICS

In the Results section of the main text we introduced a new diagnostic tool for detecting (near) degeneracy between the energy levels of molecular orbitals. The function $s_\delta(\boldsymbol{\mu})$ is defined in Equation (1) of the main text and provides a parameter δ that controls the degeneracy tolerance. We developed this metric to aid in probing the source of noncontextual discontinuities in Figure 1 of the main text, which we discovered coincide with peaks in $s_\delta(\boldsymbol{\mu})$.

In this section we supplement our new diagnostic tool with traditional approaches to detecting static correlation. For example, with $\boldsymbol{t}_1$ the vector of single-excitation amplitudes obtained from a coupled-cluster calculation, the T_1 diagnostic is defined as $||\boldsymbol{t}_1||_2/\sqrt{N_{\text{elec}}}$ [33]. It is often assumed that CCSD is reliable for $T_1 < 0.02$; however, in Figure 2 we see that T_1 never exceeds this threshold, even when CCSD becomes non-variational at 1.727Å, and thus it is not a fool-proof metric. A related diagnostic is D_1, calculated as the largest singular value of the single-excitation amplitude matrix [34] and satisfies the inequality $D_1 \geq \sqrt{2} \cdot T_1$. Correlation entropy is another quantity that can provide an indication of strong non-dynamical correlation, which is defined as the Von Neumann entropy of the natural orbital occupation numbers (NOON) $\boldsymbol{n}$,

$$\mathcal{E}(\boldsymbol{n}) = -\sum_i \frac{n_i}{2} \log\left(\frac{n_i}{2}\right), \tag{1}$$

where $0 \leq n_i \leq 2$ represents the average number of particles filling orbital i. In Figure 2 we calculate the correlation entropy with NOONs obtained from the 5-qubit contextual subspace, CCSD and FCI (exact) calculations. Above an interatomic separation of 1.494Å the CCSD-derived NOONs over-approximate the entropy and, interestingly, the entropy plateaus where the non-variational character becomes apparent. On the other hand, the NOONs obtained from the 5-qubit contextual subspace follow more closely the FCI correlation entropy. A review of these and alternative techniques can be found in [35].

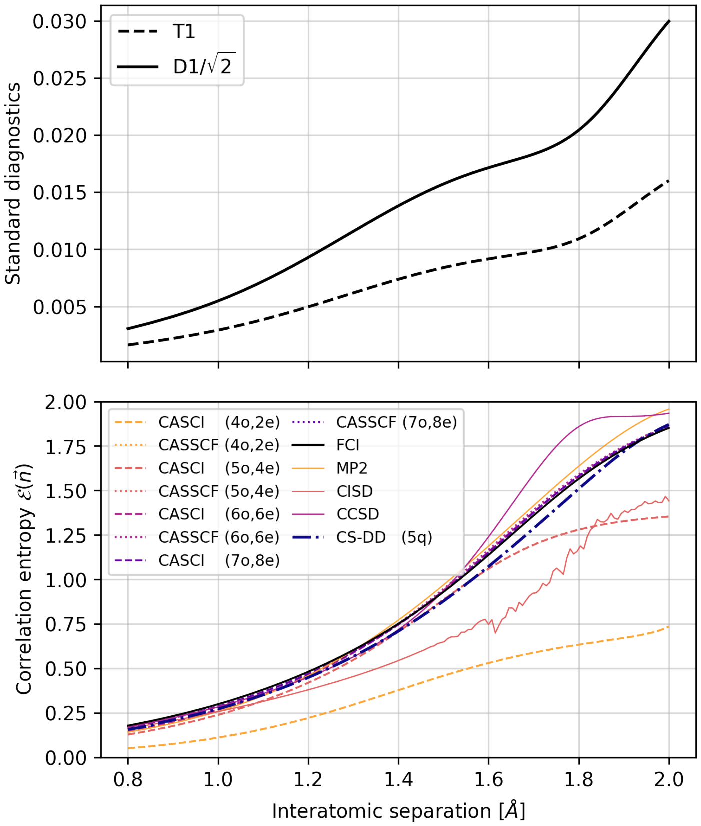

FIG. 2: A collection of typical diagnostic tools for assessing the correlations present in various wavefunction techniques.

IV. STO-3G VERSUS STO-6G

The Slater-type orbital basis set STO-nG uses $n \in \mathbb{N}$ Gaussian functions to describe each atomic orbital. In the main text above we adopted the $n = 3$ basis, however we compare here the difference between this and the $n = 6$ variant. In Figure 3, going from $n = 3$ to $n = 6$ we see the FCI potential energy curve shifted down between $28.5 - 28.9$ eV across the bond lengths $0.8 - 2$Å. Despite this large energy shift, the difference in correlation energies $(E^{(3G)}_{\text{FCI}} - E^{(3G)}_{\text{HF}}) - (E^{(6G)}_{\text{FCI}} - E^{(6G)}_{\text{HF}})$ only varies between $43 - 64$ meV; while this is small in comparison with the absolute shift, it still exceeds our target algorithmic accuracy 43 meV and therefore cannot be disregarded. In future work we will consider the use of basis sets outside of STO-3G, particularly those tailored specifically for active space methods.

V. CASCI ACTIVE SPACE SELECTION COMPARISON

In Section IV A of the main text we discussed how motivating contextual subspaces from either the MP2 or CCSD excitation generators would yield different subspaces. In Figure 2 of the main text we found that, typically, the CCSD-motivated subspaces resulted in lower errors, but at the cost of discontinuities in the potential energy curve. As such, we argued that MP2-motivated spaces were preferable, favouring more stable dissociation curves over lower subspace error.

For a fair comparison, we investigated how motivating the active space selection in CASCI via different means would affect the resulting energies. So that the methods are comparable, we used the MP2 and CCSD natural orbitals, in analogy with the contextual subspace motivation heuristic. Furthermore, we also tried simply selecting orbitals lying around the Fermi level, since naively this is where excitations are most energetically favourable.

The results of this may be viewed in Figure 4 and, surprisingly, we see a mirroring with the contextual subspace curves of Figure 2 in the main text. Indeed, the Fermi- and CCSD-motivated active spaces exhibit discontinuities, while the MP2 dissociation does not. These results motivate our use of the MP2 natural orbitals throughout the work.

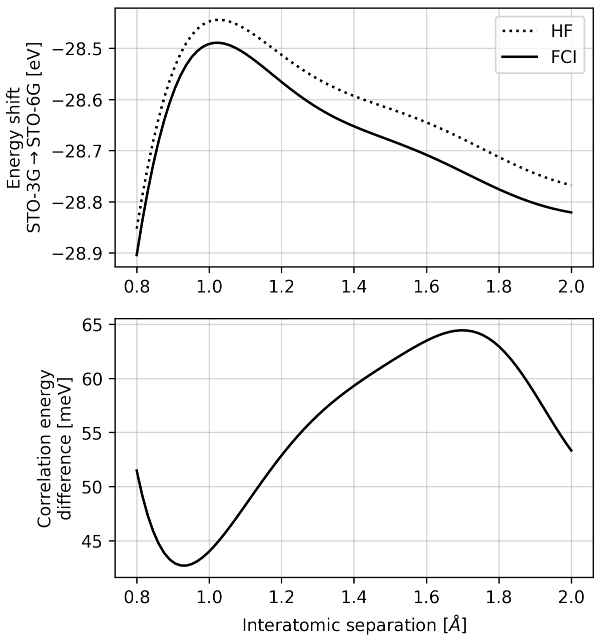

FIG. 3: Comparison of ROHF and FCI between minimal basis sets STO-3G and STO-6G. The difference in correlation energy, while small in relation to the absolute energy shift between the two basis sets, is above the target threshold of algorithmic accuracy (within 43 meV of FCI) and therefore can not be neglected.

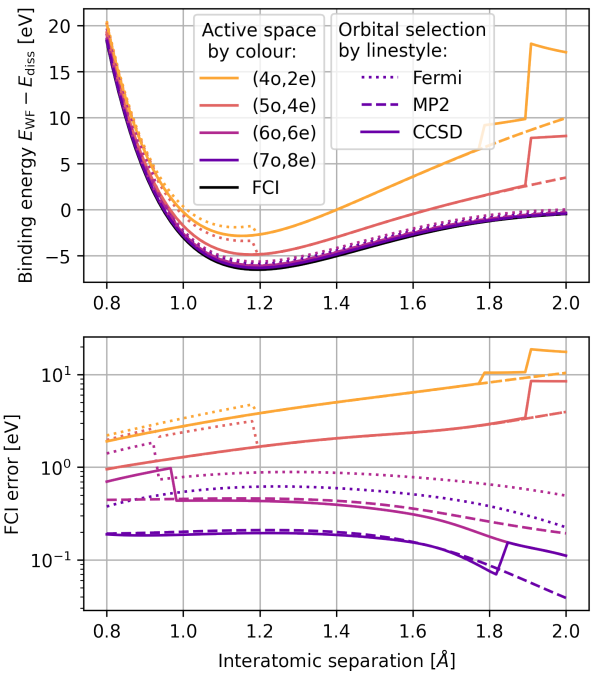

FIG. 4: Comparison of active spaces in CASCI, both varying in size and the orbital selection criteria. The sizes investigated were (4o,2e),(5o,4e),(6o,6e) and (7o,8e), while the chosen orbitals were either selected around the Fermi level or from the MP2/CCSD natural orbitals.

VI. HAMILTONIANS

TABLE II: Qubit-wise commuting decomposition of the N_2 molecular Hamiltonian at a separation of 0.80Å

**Clique Index**	**QWC Hamiltonian Terms**
Identity	$-103.58363 \cdot IIIII$
0	$+0.14413 \cdot IIIIZ + 0.14413 \cdot IIIZI + 0.64710 \cdot IIIZZ + 0.14413 \cdot IIZII + 0.28439 \cdot IIZIZ + 0.29793 \cdot IIZZI + 0.14413 \cdot IIZZZ + 0.95822 \cdot IZIII + 0.26516 \cdot IZIIZ + 0.26516 \cdot IZIZI + 0.26516 \cdot IZZII - 0.27867 \cdot IZZIZ - 0.29314 \cdot IZZZI + 0.26516 \cdot IZZZZ + 0.56212 \cdot ZIIII + 0.28110 \cdot ZIIIZ + 0.29015 \cdot ZIIZI + 0.56212 \cdot ZIIZZ + 0.23447 \cdot ZIZII - 0.25837 \cdot ZIZIZ - 0.25837 \cdot ZIZZI + 0.31482 \cdot ZIZZZ + 0.25837 \cdot ZZIII - 0.28110 \cdot ZZIIZ - 0.29015 \cdot ZZIZI + 0.25837 \cdot ZZIZZ - 0.23447 \cdot ZZZII - 0.56212 \cdot ZZZIZ - 0.56212 \cdot ZZZZI - 0.31482 \cdot ZZZZZ$
1	$+0.00905 \cdot IIXZZ + 0.01065 \cdot IXIII + 0.01065 \cdot IXIZZ + 0.02130 \cdot IXXII + 0.02130 \cdot IXXZZ + 0.01448 \cdot XIIZZ + 0.08035 \cdot XIXZZ + 0.01065 \cdot XXIII + 0.01065 \cdot XXIZZ$
2	$+0.08035 \cdot IIIYY + 0.00452 \cdot IIYYI - 0.00452 \cdot IZYYI + 0.00905 \cdot XIIYY - 0.02661 \cdot XIYIY + 0.02661 \cdot XZYIY - 0.03113 \cdot XIYYI + 0.03113 \cdot XZYYI$
3	$-0.02661 \cdot IIZXZ + 0.02661 \cdot IZZXZ + 0.02496 \cdot ZIIXZ + 0.00164 \cdot ZIZXI - 0.02496 \cdot ZZIXZ - 0.00164 \cdot ZZZXI$
4	$-0.03113 \cdot IIZZX + 0.03113 \cdot IZZZX + 0.01448 \cdot XZZZI + 0.00452 \cdot XIZZX - 0.00452 \cdot XZZZX$
5	$+0.03113 \cdot ZIIZX - 0.03113 \cdot ZZIZX + 0.00905 \cdot ZZXZI - 0.00617 \cdot ZIXZX + 0.00617 \cdot ZZXZX$
6	$+0.02130 \cdot IYYZZ + 0.08035 \cdot YIYZZ + 0.01065 \cdot YYIZZ$
7	$+0.08035 \cdot ZZZYY$

TABLE III: Qubit-wise commuting decomposition of the N_2 molecular Hamiltonian at a separation of 0.93Å

**Clique Index**	**QWC Hamiltonian Terms**
Identity	$-104.60243 \cdot IIIII$
0	$+0.17204 \cdot IIIIZ + 0.17204 \cdot IIIZI + 0.62346 \cdot IIIZZ + 0.17204 \cdot IIZII + 0.27535 \cdot IIZIZ + 0.28844 \cdot IIZZI + 0.17204 \cdot IIZZZ + 0.49343 \cdot IZIII + 0.27185 \cdot IZIIZ + 0.28110 \cdot IZIZI + 0.49343 \cdot IZIZZ + 0.21867 \cdot IZZII - 0.25019 \cdot IZZIZ - 0.25019 \cdot IZZZI + 0.30490 \cdot IZZZZ + 0.49343 \cdot ZIIII + 0.21867 \cdot ZIIIZ + 0.30490 \cdot ZIIZI + 0.49343 \cdot ZIIZZ + 0.27185 \cdot ZIZII - 0.25019 \cdot ZIZIZ - 0.25019 \cdot ZIZZI + 0.28110 \cdot ZIZZZ + 0.26913 \cdot ZZIII - 0.25643 \cdot ZZIIZ - 0.25643 \cdot ZZIZI + 0.28237 \cdot ZZIZZ - 0.25643 \cdot ZZZII - 0.96066 \cdot ZZZIZ - 0.25643 \cdot ZZZZZ$
1	$-0.02079 \cdot IIXII - 0.02079 \cdot IIXZZ - 0.01146 \cdot IXIII - 0.01146 \cdot IXIZZ + 0.08623 \cdot IXXZZ - 0.01146 \cdot XIIII - 0.01146 \cdot XIIZZ + 0.00925 \cdot XIXZZ + 0.01324 \cdot XXIZZ$
2	$-0.02079 \cdot IIIYY + 0.00925 \cdot IXIYY - 0.03386 \cdot IXYYI + 0.08623 \cdot XIIYY + 0.00463 \cdot XIYYI - 0.02923 \cdot XXYIY$
3	$-0.00925 \cdot IYIYX + 0.03386 \cdot IYXYI - 0.08623 \cdot YIIYX - 0.00463 \cdot YIXYI - 0.02923 \cdot YYXIX$
4	$+0.08623 \cdot IYYZZ + 0.00925 \cdot YIYZZ + 0.01324 \cdot YYIZZ$

TABLE IV: Qubit-wise commuting decomposition of the N_2 molecular Hamiltonian at a separation of 1.07Å

**Clique Index**	**QWC Hamiltonian Terms**
Identity	$-105.11963 \cdot IIIII$
0	$+0.19316 \cdot IIIIZ + 0.19316 \cdot IIIZI + 0.60154 \cdot IIIZZ + 0.19316 \cdot IIZII + 0.26682 \cdot IIZIZ + 0.27946 \cdot IIZZI + 0.19316 \cdot IIZZZ + 0.43837 \cdot IZIII + 0.26322 \cdot IZIIZ + 0.27266 \cdot IZIZI + 0.43837 \cdot IZIZZ + 0.20340 \cdot IZZII - 0.24369 \cdot IZZIZ - 0.24369 \cdot IZZZI + 0.29571 \cdot IZZZZ + 0.43837 \cdot ZIIII + 0.20340 \cdot ZIIIZ + 0.29571 \cdot ZIIZI + 0.43837 \cdot ZIIZZ + 0.26322 \cdot ZIZII - 0.24369 \cdot ZIZIZ - 0.24369 \cdot ZIZZI + 0.27266 \cdot ZIZZZ + 0.26015 \cdot ZZIII - 0.24902 \cdot ZZIIZ - 0.24902 \cdot ZZIZI + 0.27237 \cdot ZZIZZ - 0.24902 \cdot ZZZII - 0.97165 \cdot ZZZIZ - 0.24902 \cdot ZZZZZ$
1	$-0.02060 \cdot IIXII - 0.02060 \cdot IIXZZ - 0.01245 \cdot IXIII - 0.01245 \cdot IXIZZ + 0.09231 \cdot IXXZZ - 0.01245 \cdot XIIII - 0.01245 \cdot XIIZZ + 0.00945 \cdot XIXZZ + 0.01221 \cdot XXIZZ$
2	$-0.02060 \cdot IIIYY + 0.00945 \cdot IXIYY - 0.03671 \cdot IXYYI + 0.09231 \cdot XIIYY + 0.00472 \cdot XIYYI - 0.03198 \cdot XXYIY$
3	$-0.00945 \cdot IYIYX + 0.03671 \cdot IYXYI - 0.09231 \cdot YIIYX - 0.00472 \cdot YIXYI - 0.03198 \cdot YYXIX$
4	$+0.09231 \cdot IYYZZ + 0.00945 \cdot YIYZZ + 0.01221 \cdot YYIZZ$

TABLE V: Qubit-wise commuting decomposition of the N_2 molecular Hamiltonian at a separation of 1.20Å

**Clique Index**	**QWC Hamiltonian Terms**
Identity	$-105.37445 \cdot IIIII$
0	$+0.20897 \cdot IIIIZ + 0.20897 \cdot IIIZI + 0.58184 \cdot IIIZZ + 0.20897 \cdot IIZII + 0.25885 \cdot IIZIZ + 0.27108 \cdot IIZZI + 0.20897 \cdot IIZZZ + 0.39475 \cdot IZIII + 0.25531 \cdot IZIIZ + 0.26493 \cdot IZIZI + 0.39475 \cdot IZIZZ + 0.18898 \cdot IZZII - 0.23849 \cdot IZZIZ - 0.23849 \cdot IZZZI + 0.28736 \cdot IZZZZ + 0.39475 \cdot ZIIII + 0.18898 \cdot ZIIIZ + 0.28736 \cdot ZIIZI + 0.39475 \cdot ZIIZZ + 0.25531 \cdot ZIZII - 0.23849 \cdot ZIZIZ - 0.23849 \cdot ZIZZI + 0.26493 \cdot ZIZZZ + 0.25201 \cdot ZZIII - 0.24272 \cdot ZZIIZ - 0.24272 \cdot ZZIZI + 0.26344 \cdot ZZIZZ - 0.24272 \cdot ZZZII - 0.98735 \cdot ZZZIZ - 0.24272 \cdot ZZZZZ$
1	$-0.02060 \cdot IIXII - 0.02060 \cdot IIXZZ - 0.01353 \cdot IXIII - 0.01353 \cdot IXIZZ + 0.09837 \cdot IXXZZ - 0.01353 \cdot XIIII - 0.01353 \cdot XIIZZ + 0.00962 \cdot XIXZZ + 0.01143 \cdot XXIZZ$
2	$-0.02060 \cdot IIIYY + 0.00962 \cdot IXIYY - 0.03957 \cdot IXYYI + 0.09837 \cdot XIIYY + 0.00481 \cdot XIYYI - 0.03476 \cdot XXYIY$
3	$-0.00962 \cdot IYIYX + 0.03957 \cdot IYXYI - 0.09837 \cdot YIIYX - 0.00481 \cdot YIXYI - 0.03476 \cdot YYXIX$
4	$+0.09837 \cdot IYYZZ + 0.00962 \cdot YIYZZ + 0.01143 \cdot YYIZZ$

TABLE VI: Qubit-wise commuting decomposition of the N_2 molecular Hamiltonian at a separation of 1.33Å

**Clique Index**	**QWC Hamiltonian Terms**
Identity	$-105.73143 \cdot IIIII$
0	$-0.76333 \cdot IIIIZ - 0.04757 \cdot IIIZI + 0.03073 \cdot IIIZZ - 0.04757 \cdot IIZII + 0.03073 \cdot IIZIZ + 0.56455 \cdot IIZZI - 0.04757 \cdot IZIII + 0.03073 \cdot IZIIZ + 0.49632 \cdot IZIZI + 0.51907 \cdot IZZII - 0.04757 \cdot IZZZI + 0.03073 \cdot IZZZZ + 0.08875 \cdot ZIIII + 0.03744 \cdot ZIIIZ + 0.49635 \cdot ZIIZI + 0.51587 \cdot ZIZII + 0.08875 \cdot ZIZZI + 0.03744 \cdot ZIZZZ + 0.35124 \cdot ZZIII + 0.08875 \cdot ZZIZI + 0.03744 \cdot ZZIZZ + 0.08875 \cdot ZZZII + 0.03744 \cdot ZZZIZ + 0.55975 \cdot ZZZZI$
1	$-0.12429 \cdot IIIIX - 0.10426 \cdot IIYYI - 0.00976 \cdot IYYII + 0.00976 \cdot XIYYI + 0.07498 \cdot XYIYI - 0.08474 \cdot XYYII$
2	$+0.08474 \cdot IZZXI + 0.01088 \cdot XZZII + 0.00976 \cdot XZZXI$
3	$-0.00976 \cdot IXZZI - 0.01088 \cdot XIZZI + 0.10426 \cdot XXZZI$
4	$-0.08474 \cdot ZIZXI + 0.00976 \cdot ZXZII - 0.01097 \cdot ZXZXI$
5	$+0.10426 \cdot ZZYYI$
6	$+0.10426 \cdot YYZZI$

TABLE VII: Qubit-wise commuting decomposition of the N_2 molecular Hamiltonian at a separation of 1.47Å

**Clique Index**	**QWC Hamiltonian Terms**
Identity	$-105.94845 \cdot IIIII$
0	$-0.60340 \cdot IIIIZ - 0.03004 \cdot IIIZI + 0.02599 \cdot IIIZZ - 0.03004 \cdot IIZII + 0.02599 \cdot IIZIZ + 0.54960 \cdot IIZZI - 0.03004 \cdot IZIII + 0.02599 \cdot IZIIZ + 0.48338 \cdot IZIZI + 0.50545 \cdot IZZII - 0.03004 \cdot IZZZI + 0.02599 \cdot IZZZZ + 0.07215 \cdot ZIIII + 0.03072 \cdot ZIIIZ + 0.48359 \cdot ZIIZI + 0.50335 \cdot ZIZII + 0.07215 \cdot ZIZZI + 0.03072 \cdot ZIZZZ + 0.32684 \cdot ZZIII + 0.07215 \cdot ZZIZI + 0.03072 \cdot ZZIZZ + 0.07215 \cdot ZZZII + 0.03072 \cdot ZZZIZ + 0.54648 \cdot ZZZZI$
1	$-0.14665 \cdot IIIIX - 0.10982 \cdot IIYYI - 0.00988 \cdot IYYII + 0.00988 \cdot XIYYI + 0.08019 \cdot XYIYI - 0.09006 \cdot XYYII$
2	$+0.09006 \cdot IZZXI + 0.01053 \cdot XZZII + 0.00988 \cdot XZZXI$
3	$-0.00988 \cdot IXZZI - 0.01053 \cdot XIZZI + 0.10982 \cdot XXZZI$
4	$-0.09006 \cdot ZIZXI + 0.00988 \cdot ZXZII - 0.01078 \cdot ZXZXI$
5	$+0.10982 \cdot ZZYYI$
6	$+0.10982 \cdot YYZZI$

TABLE VIII: Qubit-wise commuting decomposition of the N_2 molecular Hamiltonian at a separation of 1.60Å

**Clique Index**	**QWC Hamiltonian Terms**
Identity	$-106.08444 \cdot IIIII$
0	$-0.48091 \cdot IIIIZ - 0.01598 \cdot IIIZI + 0.02152 \cdot IIIZZ - 0.01598 \cdot IIZII + 0.02152 \cdot IIZIZ + 0.53673 \cdot IIZZI - 0.01598 \cdot IZIII + 0.02152 \cdot IZIIZ + 0.47193 \cdot IZIZI + 0.49353 \cdot IZZII - 0.01598 \cdot IZZZI + 0.02152 \cdot IZZZZ + 0.06019 \cdot ZIIII + 0.02477 \cdot ZIIIZ + 0.47224 \cdot ZIIZI + 0.49219 \cdot ZIZII + 0.06019 \cdot ZIZZI + 0.02477 \cdot ZIZZZ + 0.30479 \cdot ZZIII + 0.06019 \cdot ZZIZI + 0.02477 \cdot ZZIZZ + 0.06019 \cdot ZZZII + 0.02477 \cdot ZZZIZ + 0.53478 \cdot ZZZZI$
1	$-0.16895 \cdot IIIIX - 0.11499 \cdot IIYYI - 0.00997 \cdot IYYII + 0.00997 \cdot XIYYI + 0.08507 \cdot XYIYI - 0.09504 \cdot XYYII$
2	$+0.09504 \cdot IZZXI + 0.01032 \cdot XZZII + 0.00997 \cdot XZZXI$
3	$-0.00997 \cdot IXZZI - 0.01032 \cdot XIZZI + 0.11499 \cdot XXZZI$
4	$-0.09504 \cdot ZIZXI + 0.00997 \cdot ZXZII - 0.01065 \cdot ZXZXI$
5	$+0.11499 \cdot ZZYYI$

TABLE IX: Qubit-wise commuting decomposition of the N_2 molecular Hamiltonian at a separation of 1.73Å

Clique Index	QWC Hamiltonian Terms
Identity	$-106.16893 \cdot IIIII$
0	$-0.38444 \cdot IIIIZ - 0.00492 \cdot IIIZI + 0.01762 \cdot IIIZZ - 0.00492 \cdot IIZII + 0.01762 \cdot IIZIZ + 0.52564 \cdot IIZZI - 0.00492 \cdot IZIII + 0.01762 \cdot IZIIZ + 0.46182 \cdot IZIZI + 0.48309 \cdot IZZII - 0.00492 \cdot IZZZI + 0.01762 \cdot IZZZZ + 0.05151 \cdot ZIIII + 0.01982 \cdot ZIIIZ + 0.46217 \cdot ZIIZI + 0.48227 \cdot ZIZII + 0.05151 \cdot ZIZZI + 0.01982 \cdot ZIZZZ + 0.28499 \cdot ZZIII + 0.05151 \cdot ZZIZI + 0.01982 \cdot ZZIZZ + 0.05151 \cdot ZZZII + 0.01982 \cdot ZZZIZ + 0.52445 \cdot ZZZZI$
1	$-0.18956 \cdot IIIIX - 0.11973 \cdot IIYYI - 0.01005 \cdot IYYII + 0.01005 \cdot XIYYI + 0.08959 \cdot XYIYI - 0.09964 \cdot XYYII$
2	$+0.09964 \cdot IZZXI + 0.01020 \cdot XZZII + 0.01005 \cdot XZZXI$
3	$-0.01005 \cdot IXZZI - 0.01020 \cdot XIZZI + 0.11973 \cdot XXZZI$
4	$-0.09964 \cdot ZIZXI + 0.01005 \cdot ZXZII - 0.01055 \cdot ZXZXI$
5	$+0.11973 \cdot ZZYYI$

TABLE X: Qubit-wise commuting decomposition of the N_2 molecular Hamiltonian at a separation of 1.87Å

Clique Index	QWC Hamiltonian Terms
Identity	$-106.22020 \cdot IIIII$
0	$-0.30710 \cdot IIIIZ + 0.00359 \cdot IIIZI + 0.01437 \cdot IIIZZ + 0.00359 \cdot IIZII + 0.01437 \cdot IIZIZ + 0.51603 \cdot IIZZI + 0.00359 \cdot IZIII + 0.01437 \cdot IZIIZ + 0.45287 \cdot IZIZI + 0.47393 \cdot IZZII + 0.00359 \cdot IZZZI + 0.01437 \cdot IZZZZ + 0.04515 \cdot ZIIII + 0.01587 \cdot ZIIIZ + 0.45323 \cdot ZIIZI + 0.47343 \cdot ZIZII + 0.04515 \cdot ZIZZI + 0.01587 \cdot ZIZZZ + 0.26725 \cdot ZZIII + 0.04515 \cdot ZZIZI + 0.01587 \cdot ZZIZZ + 0.04515 \cdot ZZZII + 0.01587 \cdot ZZZIZ + 0.51533 \cdot ZZZZI$
1	$-0.20771 \cdot IIIIX - 0.12404 \cdot IIYYI - 0.01010 \cdot IYYII + 0.01010 \cdot XIYYI + 0.09373 \cdot XYIYI - 0.10383 \cdot XYYII$
2	$+0.10383 \cdot IZZXI + 0.01016 \cdot XZZII + 0.01010 \cdot XZZXI$
3	$-0.01010 \cdot IXZZI - 0.01016 \cdot XIZZI + 0.12404 \cdot XXZZI$
4	$-0.10383 \cdot ZIZXI + 0.01010 \cdot ZXZII - 0.01047 \cdot ZXZXI$
5	$+0.12404 \cdot ZZYYI$

TABLE XI: Qubit-wise commuting decomposition of the N_2 molecular Hamiltonian at a separation of 2.00Å

Clique Index	QWC Hamiltonian Terms
Identity	$-106.87553 \cdot IIIII$
0	$-0.12236 \cdot IIIIZ - 0.12236 \cdot IIIZI + 0.11832 \cdot IIIZZ - 0.03042 \cdot IIZII - 0.01196 \cdot IIZIZ + 0.01092 \cdot IIZZI - 0.03042 \cdot IZIII + 0.01092 \cdot IZIIZ - 0.01196 \cdot IZIZI + 0.12814 \cdot IZZII + 0.12814 \cdot IZZZZ - 0.03042 \cdot ZIIII - 0.01196 \cdot ZIIIZ + 0.01092 \cdot ZIIZI - 0.01031 \cdot ZIZII - 0.01031 \cdot ZIZZZ + 0.01029 \cdot ZZIII + 0.01029 \cdot ZZIZZ - 0.01196 \cdot ZZZIZ + 0.01092 \cdot ZZZZI - 0.03042 \cdot ZZZZZ$
1	$-0.00854 \cdot IIIIX + 0.00955 \cdot IIIXI + 0.11164 \cdot IIIXX + 0.01015 \cdot IIXII + 0.00955 \cdot IIXIX - 0.00854 \cdot IIXXI + 0.01042 \cdot IXIII + 0.00854 \cdot IXIIX - 0.00955 \cdot IXIXI - 0.12794 \cdot IXXII + 0.12794 \cdot XIIII + 0.00955 \cdot XIIIX - 0.00854 \cdot XIIXI - 0.01042 \cdot XIXII - 0.01015 \cdot XXIII$
2	$-0.11164 \cdot IIIYY + 0.00955 \cdot IIYIY + 0.00854 \cdot IIYYI - 0.00854 \cdot IYIIY - 0.00955 \cdot IYIYI + 0.12794 \cdot IYYII + 0.00955 \cdot YIIIY + 0.00854 \cdot YIIYI - 0.01042 \cdot YIYII + 0.01015 \cdot YYIII$
3	$+0.12794 \cdot XZZZZ$

[1] A. Peruzzo, J. McClean, P. Shadbolt, M.-H. Yung, X.-Q. Zhou, P. J. Love, A. Aspuru-Guzik, and J. L. O'Brien, *A variational eigenvalue solver on a photonic quantum processor*, Nature communications **5**, 1 (2014).

[2] Y. Shen, X. Zhang, S. Zhang, J.-N. Zhang, M.-H. Yung, and K. Kim, *Quantum implementation of the unitary coupled cluster for simulating molecular electronic structure*, Physical Review A **95**, 020501 (2017).

[3] P. J. J. O'Malley *et al.*, *Scalable Quantum Simulation of Molecular Energies*, Physical Review X **6**, 031007 (2016).

[4] R. Santagati, J. Wang, A. A. Gentile, S. Paesani, N. Wiebe, J. R. McClean, S. Morley-Short, P. J. Shadbolt, D. Bonneau, J. W. Silverstone, D. P. Tew, X. Zhou, J. L. O'Brien, and M. G. Thompson, *Witnessing eigenstates for quantum simulation of Hamiltonian spectra*, Science Advances **4**, 1 (2018).

[5] A. Kandala, A. Mezzacapo, K. Temme, M. Takita, M. Brink, J. M. Chow, and J. M. Gambetta, *Hardware-efficient variational quantum eigensolver for small molecules and quantum magnets*, Nature **549**, 242 (2017).

[6] J. I. Colless, V. V. Ramasesh, D. Dahlen, M. S. Blok, M. E. Kimchi-Schwartz, J. R. McClean, J. Carter, W. A. de Jong, and I. Siddiqi, *Computation of Molecular Spectra on a Quantum Processor with an Error-Resilient Algorithm*, Physical Review X **8**, 011021 (2018).

[7] C. Hempel, C. Maier, J. Romero, J. McClean, T. Monz, H. Shen, P. Jurcevic, B. P. Lanyon, P. Love, R. Babbush, *et al.*, *Quantum chemistry calculations on a trapped-ion quantum simulator*, Physical Review X **8**, 031022 (2018).

[8] A. Kandala, K. Temme, A. D. Córcoles, A. Mezzacapo, J. M. Chow, and J. M. Gambetta, *Error mitigation extends the computational reach of a noisy quantum processor*, Nature **567**, 491 (2019).

[9] Y. Nam, J.-S. Chen, N. C. Pisenti, K. Wright, C. Delaney, D. Maslov, K. R. Brown, S. Allen, J. M. Amini, J. Apisdorf, K. M. Beck, A. Blinov, V. Chaplin, M. Chmielewski, C. Collins, S. Debnath, K. M. Hudek, A. M. Ducore, M. Keesan, S. M. Kreikemeier, J. Mizrahi, P. Solomon, M. Williams, J. D. Wong-Campos, D. Moehring, C. Monroe, and J. Kim, *Ground-state energy estimation of the water molecule on a trapped-ion quantum computer*, npj Quantum Information **6**, 33 (2020).

[10] S. E. Smart and D. A. Mazziotti, *Quantum-classical hybrid algorithm using an error-mitigating N-representability condition to compute the Mott metal-insulator transition*, Physical Review A **100**, 022517 (2019).

[11] A. J. McCaskey, Z. P. Parks, J. Jakowski, S. V. Moore, T. D. Morris, T. S. Humble, and R. C. Pooser, *Quantum chemistry as a benchmark for near-term quantum computers*, npj Quantum Information **5**, 99 (2019).

[12] J. E. Rice, T. P. Gujarati, M. Motta, T. Y. Takeshita, E. Lee, J. A. Latone, and J. M. Garcia, *Quantum computation of dominant products in lithium–sulfur batteries*, The Journal of Chemical Physics **154**, 134115 (2021).

[13] F. Arute, K. Arya, R. Babbush, D. Bacon, J. C. Bardin, R. Barends, S. Boixo, M. Broughton, B. B. Buckley, D. A. Buell, *et al.*, *Hartree-Fock on a superconducting qubit quantum computer*, Science **369**, 1084 (2020).

[14] Q. Gao, G. O. Jones, M. Motta, M. Sugawara, H. C. Watanabe, T. Kobayashi, E. Watanabe, Y.-y. Ohnishi, H. Nakamura, and N. Yamamoto, *Applications of quantum computing for investigations of electronic transitions in phenylsulfonyl-carbazole TADF emitters*, npj Computational Materials **7**, 70 (2021).

[15] Y. Kawashima, E. Lloyd, M. P. Coons, Y. Nam, S. Matsuura, A. J. Garza, S. Johri, L. Huntington, V. Senicourt, A. O. Maksymov, J. H. V. Nguyen, J. Kim, N. Alidoust, A. Zaribafiyan, and T. Yamazaki, *Optimizing electronic structure simulations on a trapped-ion quantum computer using problem decomposition*, Communications Physics **4**, 245 (2021).

[16] A. Eddins, M. Motta, T. P. Gujarati, S. Bravyi, A. Mezzacapo, C. Hadfield, and S. Sheldon, *Doubling the Size of Quantum Simulators by Entanglement Forging*, PRX Quantum **3**, 010309 (2022).

[17] K. Yamamoto, D. Z. Manrique, I. T. Khan, H. Sawada, and D. M. Ramo, *Quantum hardware calculations of periodic systems with partition-measurement symmetry verification: Simplified models of hydrogen chain and iron crystals*, Physical Review Research **4**, 033110 (2022).

[18] J. J. M. Kirsopp, C. Di Paola, D. Z. Manrique, M. Krompiec, G. Greene-Diniz, W. Guba, A. Meyder, D. Wolf, M. Strahm, and D. Muñoz Ramo, *Quantum computational quantification of protein–ligand interactions*, International Journal of Quantum Chemistry **122**, 1 (2022).

[19] K. Huang, X. Cai, H. Li, Z.-Y. Ge, R. Hou, H. Li, T. Liu, Y. Shi, C. Chen, D. Zheng, *et al.*, *Variational quantum computation of molecular linear response properties on a superconducting quantum processor*, The Journal of Physical Chemistry Letters **13**, 9114 (2022).

[20] P. Lolur, M. Skogh, W. Dobrautz, C. Warren, J. Biznárová, A. Osman, G. Tancredi, G. Wendin, J. Bylander, and M. Rahm, *Reference-state error mitigation: A strategy for high accuracy quantum computation of chemistry*, Journal of Chemical Theory and Computation **19**, 783 (2023).

[21] V. Leyton-Ortega, S. Majumder, and R. C. Pooser, *Quantum error mitigation by hidden inverses protocol in superconducting quantum devices*, Quantum Science and Technology **8**, 014008 (2022).

[22] Z. Liang, J. Cheng, H. Ren, H. Wang, F. Hua, Z. Song, Y. Ding, F. Chong, S. Han, Y. Shi, and X. Qian, Napa: Intermediate-level variational native-pulse ansatz for variational quantum algorithms (2023), arXiv:arXiv:2208.01215 [quant-ph].

[23] M. Motta, G. O. Jones, J. E. Rice, T. P. Gujarati, R. Sakuma, I. Liepuoniute, J. M. Garcia, and Y. Ohnishi, *Quantum chemistry simulation of ground- and excited-state properties of the sulfonium cation on a superconducting quantum processor*, arXiv preprint (2022), arXiv:2208.02414.

[24] T. E. O'Brien *et al.*, *Purification-based quantum error mitigation of pair-correlated electron simulations*, arXiv preprint (2022), arXiv:2210.10799.

[25] I. Khan, M. Tudorovskaya, J. Kirsopp, D. Muñoz Ramo, P. Warrier, D. Papanastasiou, and R. Singh, *Chemically aware unitary coupled cluster with ab initio calculations on an ion trap quantum computer: A refrigerant chemicals' application*,

The Journal of Chemical Physics **158**, 10.1063/5.0144680 (2023).
[26] L. Zhao, J. Goings, K. Shin, W. Kyoung, J. I. Fuks, J.-K. Kevin Rhee, Y. M. Rhee, K. Wright, J. Nguyen, J. Kim, *et al.*, *Orbital-optimized pair-correlated electron simulations on trapped-ion quantum computers*, npj Quantum Information **9**, 60 (2023).
[27] S. Guo *et al.*, Experimental quantum computational chemistry with optimised unitary coupled cluster ansatz (2023), arXiv:arXiv:2212.08006 [quant-ph].
[28] T. Weaving, A. Ralli, W. M. Kirby, P. J. Love, S. Succi, and P. V. Coveney, *Benchmarking noisy intermediate scale quantum error mitigation strategies for ground state preparation of the hcl molecule*, Phys. Rev. Res. **5**, 043054 (2023).
[29] P. Liu, R. Wang, J.-N. Zhang, Y. Zhang, X. Cai, H. Xu, Z. Li, J. Han, X. Li, G. Xue, *et al.*, *Performing su (d) operations and rudimentary algorithms in a superconducting transmon qudit for d= 3 and d= 4*, Physical Review X **13**, 021028 (2023).
[30] E. Dimitrov, G. Sanchez-Sanz, J. Nelson, L. O'Riordan, M. Doyle, S. Courtney, V. Kannan, H. Naseri, A. G. Garcia, J. Tricker, *et al.*, *Pushing the limits of quantum computing for simulating pfas chemistry*, arXiv preprint (2023), arXiv:2311.01242.
[31] M. A. Jones, H. J. Vallury, and L. C. Hollenberg, *Precision ground-state energy calculation for the water molecule on a superconducting quantum processor*, arXiv preprint (2023), arXiv:2311.02533.
[32] Z. Liang, Z. Song, J. Cheng, H. Ren, T. Hao, R. Yang, Y. Shi, and T. Li, *Spacepulse: Combining parameterized pulses and contextual subspace for more practical vqe*, arXiv preprint (2023), arXiv:2311.17423.
[33] T. J. Lee and P. R. Taylor, *A diagnostic for determining the quality of single-reference electron correlation methods*, International Journal of Quantum Chemistry **36**, 199 (1989).
[34] C. L. Janssen and I. M. Nielsen, *New diagnostics for coupled-cluster and møller–plesset perturbation theory*, Chemical physics letters **290**, 423 (1998).
[35] U. R. Fogueri, S. Kozuch, A. Karton, and J. M. Martin, *A simple DFT-based diagnostic for nondynamical correlation*, Theoretical Chemistry Accounts **132**, 1 (2013).
